# System for Self-excited Targeted Photodynamic Therapy Based on the Multimodal Protein DARP-NanoLuc-SOPP3

**DOI:** 10.32607/actanaturae.27331

**Published:** 2023

**Authors:** E. I. Shramova, A. Yu. Frolova, V. P. Filimonova, S. M. Deyev, G. M. Proshkina

**Affiliations:** Shemyakin-Ovchinnikov Institute of Bioorganic Chemistry, Moscow, Russian Academy of science, Moscow, 117997 Russian Federation; ”Biomarker” Research Laboratory, Institute of Fundamental Medicine and Biology, Kazan Federal University, Kazan, 420008 Russian Federation; Sechenov First Moscow State Medical University (Sechenov University), Moscow, 119991 Russian Federation

**Keywords:** bioluminescent resonance energy transfer, targeted photodynamic therapy

## Abstract

Despite the significant potential of photodynamic therapy (PDT) as a minimally invasive treatment modality, the use of this method in oncology has remained limited due to two serious problems: 1) limited penetration of the excitation light in tissues, which makes it impossible to affect deep-seated tumors and 2) use of chemical photosensitizers that slowly degrade in the body and cause photodermatoses and hyperthermia in patients. To solve these problems, we propose a fully biocompatible targeted system for PDT that does not require an external light source. The proposed system is based on bioluminescent resonance energy transfer (BRET) from the oxidized form of the luciferase substrate to the photosensitizing protein SOPP3. The BRET-activated system is composed of the multimodal protein DARP-NanoLuc-SOPP3, which contains a BRET pair NanoLuc-SOPP3 and a targeting module DARPin. The latter provides the interaction of the multimodal protein with tumors overexpressing tumor-associated antigen HER2 (human epidermal growth factor receptor type II). *In vitro *experiments in a 2D monolayer cell culture and a 3D spheroid model have confirmed HER2-specific photo-induced cytotoxicity of the system without the use of an external light source; in addition, experiments in animals with subcutaneous HER2-positive tumors have shown selective accumulation of DARP-NanoLuc-SOPP3 on the tumor site. The fully biocompatible system for targeted BRET-induced therapy proposed in this work makes it possible to overcome the following limitations: 1) the need to use an external light source and 2) the side phototoxic effect from aberrant accumulation of chemical photosensitizers. The obtained results demonstrate that the fully protein-based self-excited BRET system has a high potential for targeted PDT.

## INTRODUCTION

in oncology to treat inoperable tumors, skin and retinal cancer, as well as to irradiate the surface epithelium of organs accessible to catheters and endoscopes [1, 2, 3]. The key components of PDT are a photosensitizer (PS), excitation light of a certain wavelength, and molecular oxygen. PS photoexcitation in the presence of molecular oxygen generates singlet oxygen and/or free radicals, causing oxidative stress, followed by cell apoptosis/necrosis [4]. The obvious advantages of PDT compared to other oncology methods include low general toxicity, minimal invasiveness, and high selectivity. Low invasiveness and high selectivity are achieved by a combination of two factors: 1) the photosensitizer is activated only by light of a certain wavelength, and 2) reactive oxygen species (ROS), which have a short lifetime and thus limited diffusion in the cell, are generated in the immediate proximity to the excited PS, resulting in localized cell death. That is the reason why PDT is considered one of the most attractive photon-based methods for tumor therapy. While effectively treating the tumor, PDT remains a gentle approach in terms of its general effects on the body.

However, PDT has two significant limitations: 1) limited penetration depth (1–2 mm) of the excitation visible/near-infrared light (400–900 nm) in tissues due to light scattering by cellular structures [5], and 2) daylight-induced phototoxicity of chemical PS due to their slow biodegradation in the human body and accumulation in the skin. PS based on tetrapyrrole drugs (porphyrins and chlorins) and aminolevulinic acid approved for clinical use are known to accumulate in a patient’s tissues, causing sunlight-induce photodermatoses and hyperthermia [6, 7].

In order to address the problem of limited penetration of excitation light into the body, approaches based on the use of self-excited PDT systems are actively being developed in experimental oncology [8]. These systems are based on bioluminescence resonance energy transfer (BRET) from an oxidized form of the luciferase substrate (donor) to a PS (acceptor). A number of systems for BRET-activated PDT based on chemical PS conjugates with luciferase that demonstrated their effectiveness in *in vivo *studies have been developed over the past ten years [9, 10, 11, 12, 13, 14].

A new area of BRET-activated PDT is the development of systems based on biocompatible materials such as the ones using genetic hybrids of luciferases and protein phototoxins. Currently, only three systems demonstrating a possibility to use biomaterials for BRET-activated PDT are available.

In 2020, Kim E. et al. developed BRET-induced systems based on hybrids of RLuc luciferase and phototoxin proteins (KillerRed and miniSOG) [15]. A specific effect of the BRET-induced system on cancer cells is due to the presence of the lead peptide WLEAAYQRFL, which is specific to the integrin b1 receptor (ITGb1), in the luciferase-phototoxic protein molecule. In the absence of an external light source, the specific BRET-induced effect of this system was demonstrated in both primary tumor cells obtained from breast cancer patients and mouse xenograft tumor models.

In 2022, we proposed a fully genetically encoded BRET-induced system for PDT of deep-seated tumors [16]. The genetically engineered luciferase NanoLuc [17], used as an internal light source, and the phototoxic flavoprotein miniSOG [18], which acts as a ROS generator, were combined into one genetic construct. Using pseudotyped lentiviruses specific to the HER2 tumor marker, we have demonstrated the possibility of targeted delivery of the developed genetic construct directly inside tumor cells in the animal. We managed to inhibit the growth of both the primary tumor site and metastases. Being genetically encoded, this construct can be delivered to tumors located at any depth in the body. Later, using the phototoxic protein SOPP3 [19] (a miniSOG analogue, characterized by a high quantum yield of singlet oxygen generation), we developed a targeted system for delivery of the BRET-activated protein pair NanoLuc-SOPP3 as part of HER2-specific liposomes. We demonstrated the effectiveness of this system in both subcutaneous xenograft tumor and deep-seated disseminated intraperitoneal tumor models [20].

Apparently, the development of BRET-induced systems based on fully biocompatible and biodegradable materials allows for overcoming the problem of both excitation light delivery into deep tissues and chemical PS toxicity.

**Fig. 1:**
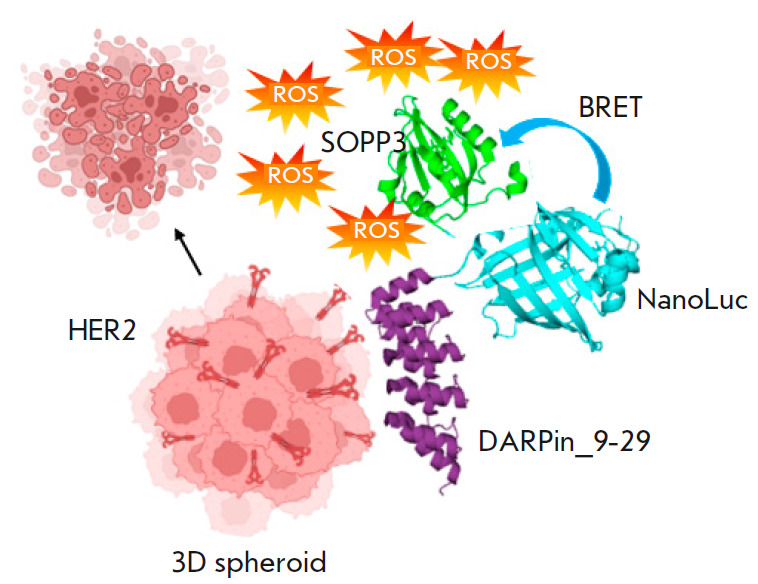
System based on the multimodular protein DARP-NanoLuc-SOPP3 for targeted BRET-induced PDT. DARPin provides binding of the NanoLuc-SOPP3 BRET pair to the HER2 receptors on the surface of cancer cells in the spheroid. Non-radiative energy transfer (BRET) from the oxidized substrate to the photosensitizing protein SOPP3 takes place in the presence of a luciferase substrate. Part of the energy is utilized for the production of reactive oxygen species (ROS), leading to cancer cell death. The illustration was generated using BioRender.com (https://www.biorender.com)

In this work, we propose the use of the multimodal targeting protein DARP-NanoLuc-SOPP3, which contains, in addition to the BRET pair NanoLuc-SOPP3, the targeting module DARPin. The latter ensures protein tropism for tumor-associated antigens of human breast and ovarian cancers. Using a 3D spheroid model, we showed that DARP-NanoLuc-SOPP3 can be used for targeted BRET-induced PDT. The experimental scheme is presented in *Fig. 1*.

## EXPERIMENTAL

**Cloning of *DARP-NanoLuc-SOPP3***

The cloning sequence of *DARPin_9-29 *was amplified from the plasmid pET22-DARP-mCherry [21] using a set of specific primers T7 forward (5’-TAA TAC GAC TCA CTA TAG GG-3’) and Dp-nano-rev (5’-GTG AAG AAG ACC ATC ATC GCG GCG CCA CCA CCA CTG CTC CCG GG-3’). The coding sequence of the NanoLuc luciferase gene was amplified from the plasmid pNL1.1.CMV (Promega) using a set of specific primers Dp-nano-dir (5’-GT GGT GGC GCG ATG GTC TTC ACA CTC GAA GAT-3’) and Nano-G4S-Bam-rev (5’-GTA CGG ATC CGC TCC CTC CGC CAC CCG CCA GAA TGC GTT CGC ACA G-3’). The 5’-regions of primers Dpnano- dir and Dp-nano-rev are mutually complementary, which allows for ligation of DARPin_9-29 and NanoLuc coding sequences during amplification. The PCR fragment encoding DARP-NanoLuc was treated with the restriction enzymes NdeI/ BamHI and cloned into the pET24 vector, pretreated with the same restriction enzymes. The *SOPP3* coding sequence was amplified from the plasmid pET24-SOPP3 (kindly provided by A.A. Pakhomov, Institute of Bioorganic Chemistry of the Russian Academy of Sciences) using the specific primers mS-Bam-dir (5’-CAT CAC GGA TCC GAA AAG AGC TTT GTG ATT ACC-3’) and mS-Hind-rev (5’- GTA CAA GCT TGC CAT CAA CCT GCA CAC CAA T-3’). The resulting PCR fragment was treated with the restriction enzymes BamHI/HindIII and ligated to vector pET24-DARP-NanoLuc, pretreated with the same set of restriction enzymes. The correct sequence of the resulting final construct was confirmed by sequinning. The coding sequence of* DARP-NanoLuc-SOPP3 *corresponds to the protein with the following primary structure:

MDLGKKLLEAARAGQDDEVRILMANGAD VNAHDFYGITPLHLAANFGHLEIVEVLLKH GADVNAFDYDNTPLHLAADAGHLEIVEVL LKYGADVNASDRDGHTPLHLAAREGHLEI VEVLLKNGADVNAQDKFGKTAFDISIDNG NEDLAEILQEFPKPSTPPGSSGGAMVFTLE DFVGDWRQTAGYNLDQVLEQGGVSSLFQN LGVSVTPIQRIVLSGENGLKIDIHVIIPYEGL SGDQMGQIEKIFKVVYPVDDHHFKVILHY GTLVIDGVTPNMIDYFGRPYEGIAVFDGKK ITVTGTLWNGNKIIDERLINPDGSLLFRVTI NGVTGWRLCERILAGGGGSGSEKSFVITDP RLPDNPIIFASDGFLELTEYSREEILGRNGR FLQGPETDQATVQKIRDAIRDQREITVQLIN YTKSGKKFLNLLNLQPIRDQKGELQAFIGV QVDGKLAAALEHHHHHH.

***DARP-NanoLuc-SOPP3 *expression**

The target protein gene was expressed in the Rosetta(DE3) *Escherichia coli *strain, transformed with plasmid pET24-DARP-NLuc-SOPP3. Transformants were grown in a liquid LB medium in the presence of kanamycin and chloramphenicol (30 and 34 μg/ml, respectively) at 37°C with aeration until the culture reached an optical density OD_600_ of 0.6–0.8. Gene expression was induced by adding isopropyl- β-D-1-thiogalactopyranoside (IPTG) to a final concentration of 0.4 mM. After addition of IPTG, the cells were grown at 37°C for 4 h and then incubated at 18°C overnight. The cell suspension was pelleted by centrifugation at 4,000 *g *for 10 min at room temperature. The resulting pellets were stored at -20°C. To isolate the protein, the cells were thawed and resuspended in wash buffer (50 mM Tris-HCl, 500 mM NaCl, and 10 mM imidazole; pH 8.0). The cells were disrupted by sonication in an ultrasonic disintegrator Sonopuls HD 3100 (Bandeline, Germany) using the following mode: ultrasonic treatment for 10 seconds and cooling for 10 seconds; 5 cycles in total. The clarified cell lysate was obtained by centrifugation at 20,000 *g *and 4°C for 30 min. The supernatant was applied to a Ni-NTA Agarose column (Qiagen) pre-equilibrated with the wash buffer. The column was washed with a 5-fold volume of wash buffer, and the protein was eluted with a buffer containing 50 mM Tris-HCl, 500 mM NaCl, and 500 mM imidazole (pH 8.0).

**Assessment of bioluminescence resonance energy transfer**

In order to evaluate the effectiveness of BRET from the donor to the acceptor in the DARP-NanoLuc- SOPP3 system, we measured the luminescence spectra of the DARP-NanoLuc-SOPP3 and NanoLuc proteins in the presence of 10 μM furimazine. Measurements were conducted using an Infinite M1000 Pro plate reader (Tecan, Austria) in the wavelength range of 400–600 nm with a step of 2 nm and an integral time of 10 ms. The BRET value was calculated as the ratio of the energy emitted by the acceptor (DARP-NanoLuc-SOPP3) to that of the donor (NanoLuc). Due to the overlap of the emission spectra of the donor and acceptor, determination of the energy transfer efficiency requires the subtraction of signals resulting from emission of the donor in the absence of the acceptor [22, 23, 24, 25]. Thus, the efficiency of energy transfer is the ratio of the donor–acceptor system (DARP-NanoLuc-miniSOG protein) emission at the wavelength of the acceptor emission maximum to the emission of the system at the wavelength of the donor emission maximum, with subtraction of the same ratio by detecting the emission spectrum of a free donor (NanoLuc protein).

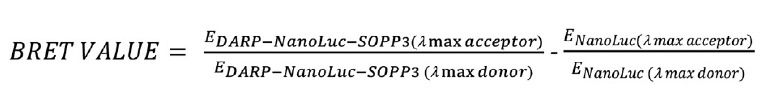



**Cell cultures**

The following cells were used in the study: HEK293T (cells that are easy to transfect) expressing the SV40 T-antigen derived from human embryonic kidney cells, SKOV3 (human ovarian carcinoma), SKOV3.ip1-Kat (cells stably expressing the far-red fluorescent protein TurboFP635), the original parental line SKOV3.ip1 (cell line isolated from intraperitoneal ascites of immunodeficient mice that were intraperitoneally injected with human ovarian adenocarcinoma SKOV3 cells), EA.hy926 (hybrid cells based on primary human umbilical vein cells and human lung adenocarcinoma A549 cells ), BJ-5TA (immortalized hTERT fibroblasts derived from human foreskin cells), and HeLa (cervical carcinoma) cells. The cells were cultured under standard conditions (in a humidified atmosphere with 5% CO_2_ at 37°C) in either a RPMI 1640 or DMEM medium (PanEco, Russia) supplemented with 2 mM L-glutamine (PanEco), 10% fetal bovine serum (Gibco), and an antibiotic (10 U/ml penicillin, 10 μg/ml streptomycin; PanEco).

**Production of cells stably expressing GFP**

seeded into a 6-well plate at a concentration of 0.6 × 10^6^ cells/ml in a complete growth medium without the antibiotic. On the day of transfection, the growth medium was replaced with a serum- and antibiotic-free medium. The third-generation lentiviral plasmids pMDLg/pPRE, pRSV-Rev, and pCMV-VSV-G, as well as the reporter plasmid pWPT-GFP, were mixed at a ratio of 2:1:0.4:2 in a serum- and antibiotic-free medium. A total of 2 μg of pMDLg/pPRE, 1 μg of pRSV-Rev, 0.4 μg of pCMV-VSV-G, and 2 μg of pWPT-GFP were added per well of a 6-well plate. The TransIntro® PL Transfection reagent (TransGen Biotech, China) was added to the DNA solution at a volume of 20 μl, mixed gently, and incubated at room temperature for 15 min. DNA–liposome complexes were added to the cells. The cells were incubated for 4–6 h. The medium was replaced with a complete culture medium. Viral particles were collected after 24, 48, and 72 h, pooled and centrifuged (10 min; 500 *g*). Viruses were added to ~70% monolayer EA.hy926 and BJ-5TA cells. The cells were centrifuged (in plates) for 90 min at 1,200 *g *in the presence of 8 μg/ml polybrene. The medium containing lentiviral particles was replaced with a fresh complete growth medium after 7 h. GFP fluorescence was assessed using an Axiovert 200M fluorescence microscope (Carl Zeiss, Germany) and flow cytometry after 72 h.

**Conjugation of DARP-NanoLuc-SOPP3 with fluorescent dyes**

SOPP3 is a weak fluorophore. In order to visualize DARP-NanoLuc-SOPP3 by flow cytometry and confocal microscopy, it was conjugated with N-hydroxysuccinimide esters of dyes (AF488 and Cy5.5; Lumiprobe, Russia). Conjugation was conducted in 20 mM phosphate buffer (pH 8.0) in the presence of a 10-fold molar dye excess. The reaction was carried out for 1 h at room temperature. The protein– dye conjugate was purified from the unreacted dye by gel permeation chromatography on a Sephadex G25 column (Cytiva).

**Flow cytometry analysis**

*GFP *expression in EA.hy926 and BJ-5TA cells after lentiviral transduction and functional activity of the HER2-specific module in the DARP-NanoLuc-SOPP3 protein were determined using flow cytometry on a NovoCyte 3000 Flow Cytometer (AceaBio, USA). For this, the cells (EA.hy926, EA.hy926-GFP, BJ-5TA, BJ-5TA-GFP, and SKOV3.ip1-Kat) were removed from the plates using Versene solution (PanEco), washed with phosphate-buffered saline, and analyzed.

To assess the ability of DARP-NanoLuc-SOPP3 to bind the HER2 receptor, the cells (HER2-positive SKOV3.ip1-Kat cells, cervical cancer HeLa cells with normal HER2 expression levels, endothelial EA.hy926 cells, and stromal BJ-5TA cells) were incubated with a 300 nM DARP-NanoLuc-SOPP3-AF488 conjugate in a complete growth medium for 10 min at 37°C. The cells were washed thrice with phosphate-buffered saline and analyzed on a NovoCyte 3000 Flow Cytometer.

GFP and AF488 fluorescence was excited by a 488 nm laser and detected in the 530 ± 30 nm channel (FITC channel).

**Confocal microscopy**

The binding of the targeting module in DARPNanoLuc- SOPP3 to the HER2 receptor on the surface of SKOV3.ip1 cells, which are characterized by overexpression of this receptor, was studied using confocal microscopy. Approximately 3,500 SKOV3.ip1 cells were seeded into 96-well glass bottom plates (Eppendorf) and cultured overnight. The 250 nM DARP-NanoLuc-SOPP3-Cy5.5 conjugate (based on the dye concentration) was added to the cells on the next day. The cells were incubated with the conjugate for 20 min and 180 min. Nuclei were stained with 10 nM Hoechst 33342 at 37°C for 10 min. The cells were washed thrice with phosphate-buffered saline, supplemented with a FluoroBrite medium (Gibco), and analyzed using an LSM 980 confocal microscope (Carl Zeiss) and a 63× Plan Apochromat oil immersion lens. A 405-nm laser was used to excite Hoechst 33342; the dye fluorescence was detected at 410–520 nm. Cy5.5 was excited with a 639-nm laser and detected at 642–755 nm.

Spheroids were analyzed using a LSM 980 confocal microscope and a ×10 dry objective in the Z-stack mode. TurboFP635 was excited with a 543-nm laser and detected in the 642- to 755-nm range. GFP was excited with a 488-nm laser and detected at 497–562 nm.

**Generation of 3D spheroids**

The 3D spheroids were grown using an anti-adhesive agarose substrate as described in [26], with modifications. In short, 81-well agarose molds were prepared from 1% of the agarose melted in phosphate-buffered saline and placed into 12-well plates. SKOV3.ip1-Kat, EA.hy926, and BJ-5TA cells were removed from the wells, washed in the culture medium, and counted. To obtain spheroids from a single cell type, 150 μl of the cell suspension containing 106 SKOV3.ip1-Kat cells were layered into each agarose mold. To obtain spheroids consisting of different cell types (epithelial, endothelial, and stromal cells), 150 μl of a suspension containing 5 × 10^5^ SKOV3.ip1-Kat cells, 2.5 × 10^5^ EA.hy926 cells, and 2.5 × 10^5^ BJ-5TA cells was layered into each agarose gel well. A complete culture medium was added to the agarose molds. The plates were centrifuged at 100 *g *for 1 min to sediment the cells to the bottom of the agarose mold. The period of spheroid formation was two days.

**Analysis of the BRET-induced cytotoxicity of DARP-NanoLuc-SOPP3 in the monolayer (2D) cell culture and spheroids (3D culture)**

To assess the BRET-induced cytotoxicity of DARP-NanoLuc-SOPP3 in the SKOV3.ip1-Kat, EA.hy926, and BJ-5TA monolayers, the cells were seeded in 96-well plates at a density of 35,000 cells/ml (SKOV3.ip1-Kat) and 25,000 cells/ml (EA.hy926 and BJ-5TA). The cells were cultured overnight under standard conditions. Different concentrations of DARP-NanoLuc-SOPP3 were added to the cells (0–1.8 μM). The cells were incubated with the protein for 20 min, and a luciferase substrate solution (30 μM) was added to the wells. The cells were incubated for 72 h under standard conditions. Cytotoxicity was analyzed using the MTT assay. The assay is based on the ability of mitochondrial dehydrogenases to convert the water-soluble tetrazolium dye 3-(4,5-dimethylthiazol- 2-yl)-2,5-diphenyltetrazolium bromide into formazan, which crystallizes in cells and has a purple color [27]. The culture medium was removed from the wells of a 96-well plate, and 100 μl (0.5 g/l) of a MTT solution (PanEco) was added to each well. The plates were incubated at 37°C for 1 h; the medium was removed, and the resulting formazan crystals were dissolved in DMSO (100 μl/well).

Spheroids grown for two days were used to assess the BRET-induced cytotoxicity of DARPNanoLuc- SOPP3 in the 3D culture. On the day of the experiment, different concentrations of DARPNanoLuc- SOPP3 (0–20 μM) were added to the cells. After 2 h of incubation, 30 μM furimazine was added into each well and the cells were incubated for 72 h under standard conditions. For cytotoxicity evaluation, each spheroid in a 10 μl agarose mold volume was placed into a well of the 96-well plate. A total of 90 μL of the MTT solution were added per well to a final concentration of 0.5 g/L. The plates were incubated at 37°C for 1 h. After the end of the incubation, the MTT solution was carefully removed using a pipette and formazan crystals were dissolved in DMSO (100 μL/well).

The absorbance of the formazan solution was measured at 570 nm using an Infinite M100 Pro plate reader (Tecan). Relative cell viability was calculated based on the ratio of absorption in experimental and control wells. The well with cells treated with 30 μM of the luciferase substrate solution was used as the control well. The DARP-NanoLuc-SOPP3 concentration causing growth inhibition of 50% of the cells in the population (IC_50_) was calculated using the GraphPad Prism software (version 9.4.0; California, USA).

**Analysis of DARP-NanoLuc-SOPP3 accumulation in HER2-positive xenograft tumor *in vivo***

The experiment on the animals was approved by the Commission of Animal Control and Welfare of the Shemyakin–Ovchinnikov Institute of Bioorganic Chemistry of the Russian Academy of Sciences (protocol 368/2022; December 19, 2022). Female Balb/c Nude mice (eight weeks old) were purchased from the Pushchino Nursery, which supplies specific pathogen- free (SPF) animals. The animals were kept in sterile conditions with unlimited access to sterile food and water.

To obtain a HER2-positive subcutaneous xenograft model, a suspension of SKOV3 cells (2 × 10^6^ cells) in a 30% Matrigel growth substrate (Corning) was inoculated subcutaneously into the right flank of mice. DARP-NanoLuc-SOPP3 biodistribution in the animal body was studied once the tumor reached 200 mm3 in volume. Approximately three weeks after tumor inoculation, 100 μl of a 40 μM DARP-NanoLuc-SOPP3-Cy5.5 conjugate (based on the dye concentration) were injected into the retro-orbital sinus. The distribution of DARPNanoLuc- SOPP3-Cy5.5 in the animal was assessed using the IVIS Spectrum In Vivo Imaging System (PerkinElmer, USA). The excitation/emission parameters for imaging were as follows: 640/680, 640/700, 640/720, 640/740, 640/760, 640/780, 675/720, 675/740, 675/760, 675/780, 710 /760, and 710/700 nm. Separation of the spectral image data was carried out using the IVIS Spectrum software.

## RESULTS AND DISCUSSION

**Production of the DARP-NanoLuc-SOPP3 protein for targeted BRET-induced PDT and its functional characterization in the 2D culture**

**Fig. 2:**
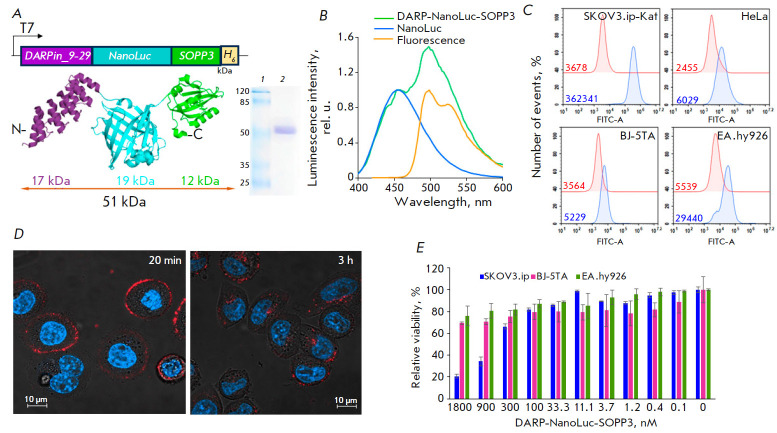
System based on the multimodular protein DARP-NanoLuc-SOPP3 for targeted BRET-induced PDT: *in vitro* characterization. (*A*) – Schematic presentation of genetic and protein DARP-NanoLuc-SOPP3 constructs. An electropherogram of a purified protein is presented to the right of the diagram. Lane 1 is a molecular weight standard; lane 2 is DARP-NanoLuc-SOPP3. (*B*) – Normalized luminescence spectra of NanoLuc (blue curve) and DARP-NanoLuc- SOPP3 (green curve) in the presence of 10 μ M furimazine. The orange curve corresponds to the normalized fluorescence spectrum of DARP-NanoLuc-SOPP3 under 460-nm laser excitation. (*C*) – receptor-specific interaction of DARP-NanoLuc-SOPP3-FITC with cells expressing the HER2 receptor at different levels. Flow cytometry data for the fluorescein 5-isothiocyanate (FITC, λ_ex_ = 488 nm, λ_em_ = 530 ± 30 nm) fluorescence channel is presented. Red lines in the pictograms correspond to fluorescently unlabeled cells (control); blue lines correspond to cells treated with DARP-NanoLuc-SOPP3-FITC. Figures in the pictograms correspond to the median fluorescence intensity. (*D*) – merged confocal images of SKOV3.ip1 cells in the blue and red fluorescence channels after incubation with DARP-NanoLuc-SOPP3-Cy5.5 for 20 minutes and 3 h. Nuclei are stained with Hoechst 33342. (*E*) – *in vitro *BRET-induced cytotoxicity of DARP-NanoLuc-SOPP3 in the presence of 30 μM furimazine. Data are presented for SKOV3.ip1, EA.hy926, and BJ-5TA cells. The scale bar in confocal images corresponds to 10 μm

To develop a targeted, fully protein-based BRETinduced system, we obtained a genetic construct encoding a targeting module specific to the HER2 tumor marker, a NanoLuc luciferase gene, and the phototoxic protein SOPP3 gene within one reading frame (*Fig. 2*). The HER2-specific protein of non-immunoglobulin scaffold DARPin_9-29, which has high affinity for the HER2 receptor (1 nM) [28], was used as a targeting molecule. HER2 is a tumor-associated antigen whose overexpression is characteristic of numerous human tumors: breast, lung, gastric, ovarian, and prostate cancers [29, 30].

The genetic construct coding DARPin- NanoLuc-SOPP3 was obtained as described in the Experimental Section (*Fig. 2A*). DARPin-NanoLuc- SOPP3 was purified by metal-chelate affinity chromatography. A denaturing polyacrylamide gel electrophoresis shows that the isolated protein has the corresponding molecular weight: 51 kDa (*Fig. 2A*). The functional activity of the phototoxic module SOPP3 in the DARPin-NanoLuc-SOPP3 hybrid construct was assessed based on the fluorescence spectrum, which fully corresponds to the published data [19] (*Fig. 2B*). BRET effectiveness of the DARPNanoLuc- SOPP3 system was determined based on the luminescent spectra of the proteins DARPNanoLuc- SOPP3 and NanoLuc obtained in the presence of the luciferase substrate furimazine (*Fig. 2B*). The resulting BRET value is 1.14, which is consistent with our previous data for NanoLuc-SOPP3 [20].

The functional activity of the DARPin targeting module in the hybrid protein was assessed based on DARP-NanoLuc-SOPP3's ability to interact with HER2 on the cancer cell surface. For this, DARP-NanoLuc-SOPP3 was conjugated to a fluorescent dye (as described in the Experimental Section). Binding of the fluorescent conjugate to HER2 was studied using flow cytometry and confocal microscopy.*Figure 2C* shows that DARP-NanoLuc-SOPP3 exhibits highly specific binding to SKOV3.ip-Kat cells characterized by HER2 overexpression, which is evidenced by a ~100-fold shift in the median fluorescence of conjugate-treated cells compared to the control. On the contrary, only a slight shift in the median fluorescence (2.5–5-fold) is observed in both epithelial HeLa cells, which are characterized by a normal HER2 expression level, and HER2-negative stromal cells (EA.hy926 and BJ5-TA) (*Fig. 2C*). Confocal microscopy showed that DARPin-NanoLuc-SOPP3-Cy5.5 effectively binds to the SKOV3.ip1 cell membrane surface during 30 min (*Fig. 2D*). Further incubation leads to DARP-NanoLuc-SOPP3 internalization. The entire protein is internalized in the cells after three hours of incubation, as evidenced by the presence of red pixels in the cytoplasmic region in the images (*Fig. 2D*). An analysis of BRET-induced cytotoxicity in the monolayer (2D) culture of HER2-positive SKOV3. ip1 cells demonstrated that DARP-NanoLuc-SOPP3 causes a phototoxic effect in the presence of furimazine with an IC_50_ of 588.6 nM, as calculated using the GraphPad Prism software.

**Functional characterization of DARP-NanoLuc-SOPP3 in the 3D culture**

The 2D models are not the optimal system for assessing drug cytotoxicity, since they do not take into account many characteristics of the tumor in the body. This is because the tumor has a three-dimensional structure; hence, such parameters as the molecular oxygen gradient, nutrients and metabolites, the presence of intercellular contacts with the cell matrix and stromal cells cannot be taken into consideration in a 2D model. It is the specific tumor microenvironment that eventually determines the metabolism heterogeneity, gene expression pattern, and, thus, the resistance of cancer cells to therapeutic drugs. Human cancer 3D models, or spheroids, provide a better platform for studying drug efficacy compared to the conventional 2D culture by reproducing important aspects of the tumor microenvironment that are the closest to *in vivo *models. This is the reason why we assessed the BRET-induced cytotoxicity of DARP-NanoLuc-SOPP3 in a culture of spheroids composed of ovarian cancer cells (SKOV3.ip1) and stroma cells presented by modified human umbilical vein EA.hy926 cells and modified fibroblast BJ-5TA cells.

**Fig. 3:**
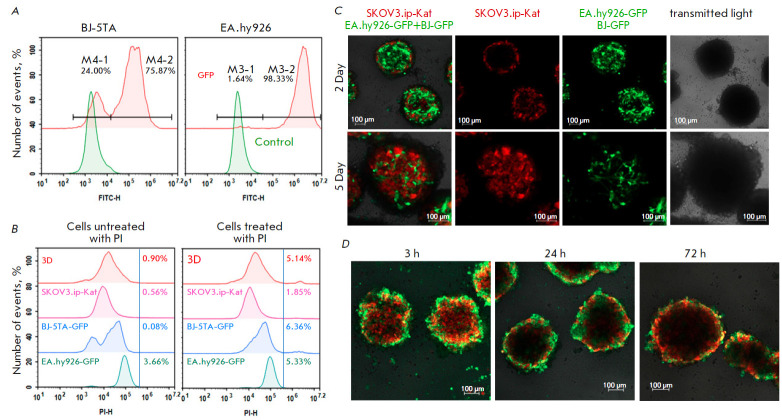
Functional characterization of DARP-NanoLuc-SOPP3 in the 3D culture. (*A*) – Efficiency of the lentiviral transduction of BJ-5TA and EA.hy926 cells with the *GFP *gene estimated by flow cytometry. Green curves correspond to non-transduced cells (autofluorescence control), red curves correspond to transduced cells. Figures correspond to the fluorescence intensity in the FITC channel (λ_ex_ = 488 nm, λ_em_ = 530 ± 30 nm) for transduced cells. (*B*) – Viability of spheroids on day 5 of cultivation. Flow cytometry data are presented for monolayer cultures (indicated in pictograms) and spheroids (3D) stained with propidium iodide (PI) (PI channel: λex = 488 nm, λem = 615 ± 20 nm). The left pictogram shows unstained cells (autofluorescence control); the right pictogram shows cells after incubation with PI. Figures correspond to the number of PI-stained cells expressed as a percentage of the total number of events. (*C*) – Confocal images of spheroids composed of SKOV3.ip-Kat, EA.hy926-GFP, and BJ-5TA-GFP cells. Merged images of spheroids in the red and green fluorescence channels (left column) and separate spheroid images in the red and green fluorescence channels on days 2 and 5 of cultivation are shown. The right column corresponds to the image of the spheroids in the transmitted light. (*D*) – DARP-NanoLuc-SOPP3-FITC interaction with the spheroids of SKOV3.ip-Kat, EA.hy926, and BJ-5TA cells. Confocal images of the spheroids after incubation with DARP-NanoLuc-SOPP3-FITC (0.5 μM, based on the dye concentration) for 3, 24, and 72 h, respectively, are presented. The scale bar in the confocal images corresponds to 100 μm

In order to analyze the spheroid structure by confocal microscopy, EA.hy926 and BJ-5TA cells stably expressing *GFP *were generated using lentiviral transduction. The transduction efficiency was evaluated using flow cytometry. *Figure 3A* shows that the *GFP* transduction level in EA.hy926 and BJ-5TA cells was 98.33 and 75.87%, respectively.

Cell viability in the spheroids was determined by estimating the number of dead cells in the culture using propidium iodide staining and flow cytometry. SKOV3.ip1-Kat, EA.hy926-GFP, and BJ-5TA-GFP cell spheroids were used in the experiment. The original cell lines were used as controls. Spheroids were lysed using trypsin on day 5 of growth and then stained with propidium iodide. Fluorescence was measured in the propidium iodide channel. *Figure 3B* demonstrates that the number of dead cells in the spheroids is similar to that of dead cells in the original cell cultures on the corresponding day of cultivation with the same number of analyzed cells.

The spheroids were formed as described in the Experimental Section. *Figure 3C* shows that the resulting structures have the morphology of spheroids with a developed stromal network (green strands). Spheroids significantly increase in volume during cul tivation; by day five of cultivation, stromal cells are almost completely covered by ovarian adenocarcinoma cells, which is in complete agreement with the previously published data on spheroids of a similar composition [31].

To study the interaction of DARP-NanoLuc-SOPP3 with spheroids, we obtained the spheroids of fluorescent SKOV3.ip-Kat cells, as well as EA.hy926 and BJ-5TA cells not modified with GFP. To visualize the interaction of DARP-NanoLuc-SOPP3 with the HER2 receptor on the spheroid surface, the DARP-NanoLuc-SOPP3–FITC conjugate was used. *Figure 3D* shows that DARP-NanoLuc-SOPP3-FITC effectively interacts with the spheroids, as indicated by the presence of the green “crown” around the spheroid. The green fluorescent signal on the spheroid surface decreases with time, indicating that DARP-NanoLuc-SOPP3 has penetrated into the spheroid.

**Fig. 4:**
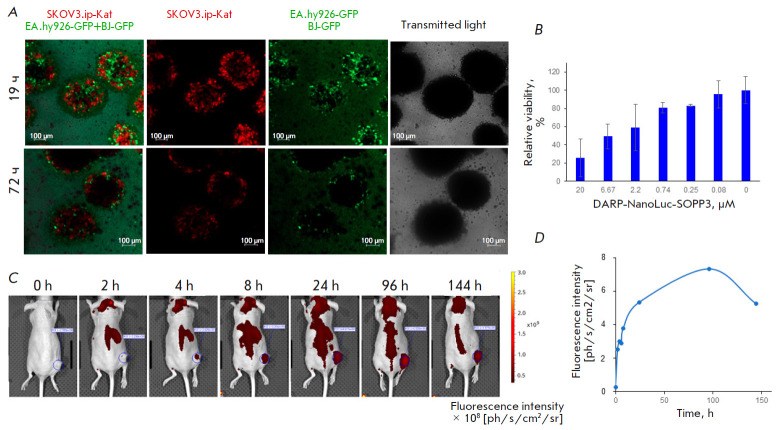
DARP-NanoLuc-SOPP3 BRET-induced cytotoxicity in the 3D culture and biodistribution *in vivo*. (*A*) **– **Confocal images of spheroids (SKOV3.ip-Kat, EA.hy926-GFP, and BJ-5TA-GFP cells) after incubation with DARP-NanoLuc- SOPP3 (300 μM) for 19 and 72 h. (*B*) – BRET-induced cytotoxicity of DARP-NanoLuc-SOPP3 in spheroids composed of ovarian cancer adenocarcinoma and stromal cells. (*C*) – Distribution of DARP-NanoLuc-SOPP3-Cy5.5 in a mouse with subcutaneous HER2-positive SKOV3 tumor (encircled by a blue dotted line). (*D*) – Dependence of the fluorescence intensity (expressed in photons per second per cm^2^ per steradian) on time. The graph is based on data corresponding to the average brightness of the tumor area in *Fig. C *(blue dotted line) at a certain time point

The BRET-induced phototox i c i t y o f DARP-NanoLuc-SOPP3 was studied in the spheroids of SKOV3.ip-Kat, EA.hy926-GFP, and BJ-5TA-GFP cells. *Figure 4A* demonstrates a change in the spheroid structure morphology over time under the effect of DARP-NanoLuc-SOPP3 in the presence of furimazine. We would like to note that, in the interval of 19–72 h, the fluorescent signal decreases for both HER2-positive SKOV3.ip-Kat adenocarcinoma and stromal cells. The BRET-induced cytotoxicity of DARP-NanoLuc-SOPP3 in the presence of furimazine was ~10 times lower in the 3D culture compared to the 2D culture: the IC_50_ was 6.58 μM, as calculated using the GraphPad Prism v. 10.1.0 software (*Fig. 4B*).

To study the role of the DARPin targeting module in the selective accumulation of DARP-NanoLuc- SOPP3 in HER2-positive animal tumors, the pattern of the DARP-NanoLuc-SOPP3–Cyanine 5.5 conjugate accumulation was studied in the tumor. For this, we used mice with subcutaneous xenograft tumors based on SKOV3 cells. The IVIS Spectrum In Vivo Imaging System was used for imaging. The fluorescent signal is first detected in the tumor two hours after intravenous injection of a 40-μM DARP-NanoLuc- SOPP3-Cy5.5 solution (based on the dye concentration) to the animals. The signal gradually increases, reaching its maximum after 96 h (*Fig. 4C,D*), and then decreases. The obtained results indicate that the DARPin targeting module in the NanoLuc-SOPP3 BRET pair not only allows for rapid (within the first 2–4 h after injection) accumulation of the drug in the tumor, but also makes it possible to avoid its accumulation in vital organs.

## CONCLUSION

Conventional PDT is a very promising approach in cancer treatment given its spatial and temporal selectivity, as well as minimal invasiveness of healthy cells. However, the limited depth of light penetration required for PS activation, as well as aberrant accumulation of chemical PS in skin cells leading to undesira ble light-induced side effects, hinders the widespread clinical use of PDT [5, 32]. The development of BRETactivated systems based on completely biocompatible components can help solve these problems.

HER2-specific BRET-activated PDT based on the multimodal protein DARP-NanoLuc-SOPP3, which consists of a HER2-secific targeting module and a NanoLuc-SOPP3 protein pair for BRET-induced PDT. *In vitro *experiments and experiments in the 3D spheroid model confirmed the photo-induced cytotoxicity of the system in HER2-positive human ovarian adenocarcinoma cells without the need for an external light source. Moreover, experiments in animals carrying subcutaneous HER2-positive tumors demonstrated selective accumulation of DARPNanoLuc- SOPP3 at the tumor site. Considering the available data on the half-life of the luminescent signal in the NanoLuc–furimazine system, which is > 2 h [17], as well as our data on the BRET-induced phototoxicity of NanoLuc-SOPP3 [20], we believe that the multimodal protein DARP-NanoLuc-SOPP3 can be used for *in vivo *therapy. The regimen for administration of the protein and luciferase substrate furimazine, which ensures long-term, simultaneous, and high maintenance of these components in the tumor, which are key for BRET-induced PDT, is proposed.

Our results show that there is great potential in the developed protein targeted self-exciting BRET system for PDT.

Based on the conducted experiments, we can conclude that the fully biocompatible system for targeted BRET-induced therapy using DARP-NanoLuc-SOPP3 makes it possible to overcome the two major limitations of conventional PDT: 1) the side phototoxic effect from the aberrant accumulation of chemical PS that results in light-activated responses, and 2) the need to use an external light source, which can often be achieved only by using expensive high-tech devices.

## Abbreviations

BRET: bioluminescent resonance energy transfer
HER2: human epidermal growth factor receptor type II
PDT: photodynamic therapy
PS: photosensitizer
